# Optimizing care for patients who have ascites: shifting paracentesis from the emergency department to outpatient medicine

**DOI:** 10.1007/s43678-026-01095-5

**Published:** 2026-01-28

**Authors:** Laurent Dubé, Francis Dubé, Celine Fresne

**Affiliations:** 1 Hôpital Montfort, University of Ottawa, Ottawa, ON Canada; 2 Hôpital Montfort, Institut du Savoir Montfort, University of Ottawa, Ottawa, ON Canada

Dear Editor,

We would like to address the current care pathways for patients requiring paracentesis for ascites and highlight opportunities to improve and streamline their management. Herein, we report a retrospective study in the Emergency Department (ED) of Hôpital Montfort, Ottawa, Ontario conducted between April 2022 and April 2024, concluding most paracenteses performed in the ED could likely have been safely scheduled and performed in an outpatient setting, thereby improving patient experience while reducing strain on hospital resources. The study received a compliance letter from the Hôpital Montfort Research Ethics Board.

Among the 32 patients included (46 visits), 75% had a known history of cirrhosis, and 78% had access to a primary care provider. Despite this, 60% of visits involved patients with a prior history of paracentesis, and nearly two-thirds of presentations resulted in hospital admission. Twenty-six of the 46 visits (57%) were single visits, while the remainder were recurrences, including one patient with seven visits. Thus, 43% of paracenteses were performed in six patients. Almost half of the visits occurred outside regular clinic hours, suggesting that patients lacked alternative access points for timely evaluation and management of symptomatic ascites.

These findings informed the development of criteria to identify patients who may be appropriate candidates for scheduled outpatient paracentesis. The key determinants include hemodynamic stability, absence of clinical suspicion for spontaneous bacterial peritonitis, and a well-established diagnosis of chronic ascites. We also proposed criteria for recognizing potentially avoidable ED visits, such as non-acute decompensation of known cirrhosis and if patients are already followed by hepatology or primary care.

Our analysis also supports three main recommendations aimed at reducing unnecessary ED utilization: 1) implementing a structured patient education program, 2) strengthening coordination between the ED and the hepatology clinic—including rapid and direct access to clinic scheduling—and 3) enhancing collaboration with primary care providers. These initiatives would help ensure timely outpatient access to paracentesis and improve continuity of care for this chronically ill population.

Given the growing burden of liver disease and the considerable resource implications of ascites-related ED visits and admissions, it is essential to rethink current care pathways. Studies showed outpatient paracentesis models, using nurse-led care or telemedicine, reduce ED use, improve access, and do not increase adverse events [1–4]. Prospective validation of our proposed criteria is a necessary next step toward establishing an efficient model that safely transitions appropriate paracenteses away from the ED and into the outpatient setting.

## Data Availability

Data are available upon request to the corresponding author.
